# Emergency management of decompensated peripartum cardiomyopathy

**DOI:** 10.4103/0974-2700.50748

**Published:** 2009

**Authors:** Indu Lata, Renu Gupta, Sandeep Sahu, Harpreet Singh

**Affiliations:** Department of Obstetrics and Gynaecology V.M.M.C and Safdarjung Hospital, N. Delhi, India; 1 Department of Obstetrics and Gynaecology, G.S.V.M. Medical College, Kanpur, India; 2 Department of Anaesthesiology, G.S.V.M. Medical College, Kanpur, India

**Keywords:** Heart failure, peripartum cardiomyopathy, pre-eclamptic toxemia, pregnancy, pulmonary edema

## Abstract

Peripartum cardiomyopathy (PPCM) is a rare life-threatening cardiomyopathy of unknown cause that occurs in the peripartum period in previously healthy women.[1] the symptomatic patients should receive standard therapy for heart failure, managed by a multidisciplinary team. The diagnosis of PPCM rests on the echocardiographic identification of new left ventricular systolic dysfunction during a limited period surrounding parturition. Diagnostic criteria include an ejection fraction of less than 45%, fractional shortening of less than 30%, or both, and end-diastolic dimension of greater than 2.7 cm/m^2^ body surface-area. This entity presents a diagnostic challenge because many women in the last month of a normal pregnancy experience dyspnea, fatigue, and pedal edema, symptoms identical to early congestive heart failure. There are no specific criteria for differentiating subtle symptoms of heart failure from normal late pregnancy. Therefore, it is important that a high index of suspicion be maintained to identify the rare case of PPCM as general examination showing symptoms of heart failure with pulmonary edema. PPCM remains a diagnosis of exclusion. No additional specific criteria have been identified to allow distinction between a peripartum patient with new onset heart failure and left ventricular systolic dysfunction as PPCM and another form of dilated cardiomyopathy. Therefore, all other causes of dilated cardiomyopathy with heart failure must be systematically excluded before accepting the designation of PPCM. Recent observations from Haiti[2] suggest that a latent form of PPCM without clinical symptoms might exist. The investigators identified four clinically normal postpartum women with asymptomatic systolic dysfunction on echocardiography, who subsequently either developed clinically detectable dilated cardiomyopathy or improved and completely recovered heart function.

## INTRODUCTION

PPCM is a rare and potentially fatal disease in pregnant, mortality rate of peripartum cardiomyopathy is 30%–60% and may be caused by severe pulmonary congestion and/or thrombo-embolic events. Survivors have a 50%–80% risk of developing cardiac failure during future pregnancies, with an associated mortality rate of 60%; so only timely diagnoses and prompt management can save two lives.

## CASE REPORT

A 37-year-old female (primigravida) G1P0L0 presented to the emergency department at night at 37 weeks gestation with chief complaint of progressively increasing breathlessness for 15 days and swelling in both lower limbs for 7 days. Routine general and obstetrical assessment showed on examination: general condition – poor, blood pressure – 180\110 mmHg, pulse – 136\min irregular, respiratory rate – 36\min, Pallor ++, JVP raised, pedal edema + cardiovascular exam showed S3 gallop rhythm present, P2 loud (pulmonary hypertension) and chest with bilateral crepitations (pulmonary edema). The patient's presenting complaints and examination indicated the presence of a cardiovascular system problem. Echocardiography performed demonstrated: EF 30% with mild generalized hypokinesia of left ventricle, mild to moderate TR and mild MR with signs of congestive heart failure (CHF). She was diagnosed with peripartum cardiomyopathy (PPCM) with left ventricular failure (EF 30%) with severe pre-eclampsia (PET). Immediate medical management was initiated with fruselac 50 mg, M-Dopa 200 mg QID, amlodipine 5 mg OD, deriphyllin 1 tab BID, and nebulization therapy every 8 h, Ensuing blood pressure was 170\106 mmHg at which time a nitroglycerine infusion was started. When the patient developed abdominal pain the next morning with thick meconium, she underwent an emergency Caesarean section. Echocardiography demonstrated an ejection fraction of 30% with mild generalized hypokinesia of left ventricle, mild to moderate TR and mild MR with signs of congestive heart failure (CHF). Her preoperative laboratory investigation showed hemoglobin of 10.2 gm%, B+ blood group, and unremarkable liver and kidney functions, coagulation profile, and normal blood sugar. A central venous pressure line was placed to manage the fluid overload. The initial CVP was 20 mmHg. The patient's congestive heart failure was managed with digoxin, dobutamine, and nitroglycerine infusion. Ranitidine and metoclopramide were given because the patient had not been on NPO status. Rapid sequence induction was performed utilizing etomidate: after delivery of the baby, oxytocin was started. In the post-operative state, pink frothy sputum was noted from the endotracheal tube. She was treated for pulmonary edema with fruselac(lasix) 40 mg, increased nitroglycerine, dobutamine, and ventilator support with controlled mode ventilation with positive end expiratory pressure (CMV+PEEP). The patient was managed with incremental lasix given up to 100 mg with hemodynamic monitoring drip of dobutamine and nitroglycerine, digoxin , and low molecular weight heparin (daltaparin 2500 IU). With aggressive management on the second hospital day, the patient's clinical status improved, and she was successfully extubated the next morning (3^rd^ hospital day). The patient was maintained on oral digoxin, ramipril, lasix, and potassium chloride. Oral atenolol was added on the 5th hospital day and she was transferred to a general obstetric ward on the 7th hospital day. On the 8th day after confinement, echocardiography showed improvement in ejection fraction to 35%. She was discharged with a healthy baby to home on the 12th day on digoxin, ramipril, potassium chloride, and a diet of salt restriction.

## DISCUSSION

The clinical presentation on patients with peripartum cardiomyopathy is similar to that of patients with systolic heart failure.[1] the left ventricular ejection fraction and the left ventricular end-diastolic diameter are significant prognostic factors. In the absence of systematic studies, comparing therapeutic approaches in PPCM, standard heart failure therapy (diuretics, vasodilators, and digoxin, anticoagulant as needed) should be initiated. Careful attention must be paid to fetal safety and to excretion of drug or drug metabolites during breastfeeding after delivery.

Therapy regimens include diuretics to diminish volume overload (preload) after load reduction with angiotensin-converting-enzyme inhibitors (postpartum only) and beta-blockers after signs and symptoms of congestion have improved. ACE inhibitors should be considered a mainstay of treatment for PPCM after delivery. Treatment options during pregnancy include hydralazine and nitrates, and we started nitroglycerine from beginning when patient came. Calcium channel blockers can be used during pregnancy to control blood pressure (and decrease uterine contractility), but most have negative inotropic properties.[3]

Second-generation beta-adrenoreceptor antagonists have beneficial effects in selected patients with dilated cardiomyopathy in the postpartum period in patients who continue to have symptoms and echocardiographic evidence of left ventricular compromise despite more than 2 weeks of standard heart failure management.

Patients with significantly depressed left ventricular function (ejection fraction ≤ 35%) may benefit from anticoagulation therapy to prevent thrombosis and emboli. Arrhythmias should be treated according to standard protocols. Atrial arrhythmias may be treated with digoxin, which may also be used for its positive inotropic effect. Salt and water restriction are important, particularly in women with symptoms and signs of heart failure. If this treatment is ineffective, more aggressive ventricular support such as intra-aortic balloon counter pulsation, and left ventricular assist device or heart transplantation may be considered.

When cardiomyopathy occurs during pregnancy, delivery of the fetus reduces the hemodynamic stresses on the heart. As cardiomyopathy manifests in the final trimester, the fetus is usually mature and can be delivered safely before or at the time of commencement of medical therapy. The mode of delivery for patients with peripartum cardiomyopathy is generally based on obstetric indications. The advantages of vaginal delivery are minimal blood loss, greater hemodynamic stability, avoidance of surgical stress, and less chance of postoperative infection and pulmonary complications. Effective pain management is a necessity to avoid further increases in cardiac output from pain and anxiety. The use of local infiltration anesthesia in combination with bilateral ilio-inguinal nerve block has been described. However, regional anesthesia has the additional advantages of reducing preload and after load and minimizes the fluctuations in cardiac output associated with labor. Regional anesthesia is contraindicated in anticoagulated patients. Caesarean delivery is reserved for indications such as fetal distress or failure to progress as in our case.

There is scant information in the literature regarding the anesthetic management of peripartum cardiomyopathy, although several anesthetic options for CS have been reported.[2,4] In patients undergoing general anesthesia, the principles of anesthetic management are as for any patient with cardiac failure: maintenance of normal to low heart rate to decrease oxygen demand, and prevention of large swings in blood pressure. However, performing a rapid sequence induction on a patient with compromised cardiac function can be very challenging. The hypertensive response to intubation should be minimized; in the above instance we used esmolol, fentanyl and Xylocard before careful titration of the induction agent. Careful hemodynamic monitoring and fluid balance is obligatory and arterial and CVP lines are recommended prior to surgery. Early critical care referral is essential for unstable patients and critically ill patients with pulmonary edema, hypoxia, mental obtundation, hypotension, refractory oliguria or acidaemia will require Swan–Ganz monitoring, artificial ventilation and inotropic support. Outcome is dependent on the ejection fraction and left ventricular end-diastolic volume at diagnosis, response to medical therapy, and normalization of left ventricular function within 6 months of pregnancy.

## OVERVIEW

The incidence of peripartum cardiomyopathy ranges from 1 in 1300 to 1 in 15,000 pregnanccies.[1] Peripartum cardiomyopathy is a rare and under recognized form of dilated cardiomyopathy, defined as heart failure in the last month of pregnancy or in the first five months post-partum with absence of determinable cause for cardiac failure and absence of demonstrable heart disease.[5] Cardiovascular status may benefit from prompt vaginal delivery or Caesarean Section.[6] There is an increased incidence with multiple gestation, pre-eclampsia, obesity, advanced maternal age, African descent and prolonged tocolysis.[7] The etiology is uncertain, viral, autoimmune and idiopathic etc. have been considered.[3] Cardiomyopathy is usually a diagnosis of exclusion. The prognosis is related to the recovery of ventricular function. The mortality rate of peripartum cardiomyopathy is 30%–60% and may be caused by severe pulmonary congestion, and/or thrombo-embolic events.[4–7] Common misdiagnoses include other types of cardiomyopathy, valvulopathies, accelerated hypertension, diastolic ventricular dysfunction, systemic infection and pulmonary embolism.[8] If supportive treatment fails, cardiac transplantation may be indicated.[9] Survivors have a 50%-80% risk of developing cardiac failure during future pregnancies, with an associated mortality rate of 60%.[10] Peripartum cardiomyopathy (PPCM) is a disorder in which initial left ventricular systolic dysfunction and symptoms of heart failure occur between the late stages of pregnancy and the early postpartum period. It is common in some countries and rare in others. The causes and pathogenesis are poorly understood. Molecular markers of an inflammatory process are found in most patients. Clinical presentation includes usual signs and symptoms of heart failure, and unusual presentations relating to thromboembolism. Clinicians should consider PPCM in any peripartum patient with unexplained disease. Conventional heart failure treatment includes use of diuretics, beta blockers, and angiotensin-converting enzyme inhibitors. Effective treatment reduces mortality rates and increases the number of women who fully recover left ventricular systolic function. Outcomes for subsequent pregnancy after PPCM are better in women who have first fully recovered heart function. Areas for future research include immune system dysfunction, the role of viruses, nonconventional treatments such as immunosuppression, immunoadsorption, aphaeresis, antiviral treatment, suppression of proinflammatory cytokines, and strategies for control and prevention.[11]

## EPIDEMIOLOGY

Although it seems likely that women of reproductive age all over the world have some risk of developing PPCM, good data about incidence are unavailable because so few population-based registries exist. Recent reports suggest an estimated incidence of one case per 299 live births in Haiti[12,13] one case per 1000 live births in South Africa, and one case per 2289 live births to one case per 4000 live births in the USA.[14] These more recent data from the USA suggest a higher incidence than reported in earlier studies.[14,15] The reasons for this variation in incidence between countries remain unknown. Cases of PPCM that adhere to the stated diagnostic criteria probably represent a similar disease, despite geographical variation in contributing factors. The remarkable propensity for recovery of left ventricular function observed in the diverse locations provides further evidence for a similar disease process. It should not be surprising that varying genetic pools and diverse environmental factors play parts of varying importance.

## PATHOPHYSIOLOGY

Pregnancy stresses the cardiovascular system, often worsening known heart disorders or unmasking occult heart disorders. Stresses include decreased hemoglobin and increased blood volume, stroke volume, and eventually, heart rate. Cardiac output increases by 30% to 50%. These changes become maximal between 28 and 34 weeks gestation. During labor, cardiac output increases approximately 20% with each uterine contraction; other stresses include straining during the 2^nd^ stage of labor and the increase in venous blood returning to the heart from the contracting uterus. Cardiovascular stresses do not return to pre-pregnancy levels until several weeks after delivery. The cause and mechanism of pathogenesis of PPCM remain unknown, and many hypotheses have been proposed. Early suggestions that nutritional disorders, such as deficiencies in selenium and other micronutrients, might contribute could not be confirmed in studies of Haitian patients with PPCM, although unidentified nutritional factors might exist.

Because of immune system changes related to pregnancy, associations with autoimmune mechanisms and inflammation have been studied since the 1970s. Melvin and colleagues[16] proposed myocarditis as the cause for PPCM and reported a dense lymphocyte infiltrate with variable amounts of myocyte edema, necrosis, and fibrosis in right ventricular biopsy specimens. Treatment with prednisone and azathioprine resulted in clinical improvement and loss of inflammatory infiltrate on repeated biopsies in the three patients studied. Sanderson and colleagues[17] and subsequently Midei and colleagues,[18] emphasized the association between myocarditis and development of PPCM. Although their findings were intriguing, they failed to establish a causal link. Additionally, Rizeq and colleagues[19] found an inflammatory component in less than 10% of biopsy samples from patients with PPCM, a proportion similar to that found in age-and-sex-matched patients with idiopathic dilated cardiomyopathy. A decade later, Felker and colleagues[21] confirmed that the absence or presence of inflammation on endomyocardial biopsy tissue did not predict outcome in patients with PPCM. A viral trigger for the development of PPCM has been postulated and investigated in several studies. Although various causes for PPCM have been proposed, including abnormal hormonal regulation, the role of innate and adaptive immune systems and the participation of auto antibodies, progenitor dendritic cells, T and B lymphocytes, cytokines, and chemokines,[31] so far no cause has been clearly identified. It is likely that the etiology is multifactorial, and that the search for a cause is hampered by the rarity of this condition in the developed countries, where more research funds are available, by the lack of adherence to diagnostic guidelines, and by the heterogeneity of the populations studied.

## CLINICAL PRESENTATION

The most common presentation of PPCM is with symptoms and signs of systolic heart failure.[21,22] Clinical examination of 97 patients seen in South Africa over 4 years revealed a displaced hypodynamic apical impulse in 72% and gallop rhythm in 92% of the patients. Functional mitral regurgitation was present in 43%. Left ventricular hypertrophy by electrocardiographic voltage criteria was present in 66% and ST-T wave abnormalities in 96% of the patients. Additional symptoms and signs include dependent edema, dyspnea on exertion, orthopnoea, paroxysmal nocturnal dyspnea, persistent cough, abdominal discomfort secondary to passive congestion of the liver and other organs, precordial pain, and palpitations. In the later stages, postural hypotension may be prominent, reflecting low cardiac output and low blood pressure.

Early signs and symptoms of heart failure can be obscured by pregnancy, because often the patient considers them to be a normal part of pregnancy. New York Heart Association (NYHA) cardiac functional classification varies from NYHA I to IV, although most frequently the initial presentation is with NYHA III and IV. Sudden cardiac arrest might occur in a situation where cardiopulmonary resuscitation is neither available nor successful.[23] Delayed diagnosis may be associated with increased morbidity and mortality. A broad index of suspicion in any peripartum patient with unexplained disease is important since unusual and non-heart failure presentations are also possible, as illustrated by the following evidence.

Left ventricular thrombus is common in PPCM patients with a left ventricular ejection fraction of less than 0·35. With progression of disease, four-chamber dilation may be seen, with thrombus formation in the left atrium and right ventricle. Peripheral embolisation then becomes possible to any part of the body, including arterial occlusion of the lower extremities with compromised circulation,[24,25] cerebral embolism with hemiplegia, aphasia, and incoordination[26] mesenteric artery occlusion with an acute abdomen secondary to bowel infarction and acute myocardial infarction secondary to coronary artery embolism.[27,28] Hemoptysis may be the presenting feature of co-existing pulmonary embolus, for which the patients also have an increased risk.

## MANAGEMENT AND PROGNOSIS

The medical management of patients with PPCM is similar to that for other forms of heart failure, and has been reviewed in detail.[29] Treatment aims to reduce after load and preload, and to increase contractility. Angiotensin-converting enzyme (ACE) inhibitors are usually used to reduce after load by vasodilatation if PPCM occurs after pregnancy. Because of potential toxic effects on the fetus, hydralazine (with or without nitrates) should replace ACE inhibitors during pregnancy. Beta blockers are used since high heart rate, arrhythmias, and sudden death often occur in patients with PPCM. Digitalis, an inotropic agent, is also safe during pregnancy and may help to maximize contractility and rate control, but serum levels have to be closely monitored since excessive digoxin concentrations in serum have been associated with worse outcomes in women.[30,31] Women in general, and pregnant women in particular, may be more sensitive than men to its effect. Diuretics are also safe and are used to reduce preload and relieve symptoms. Because of the high incidence of thromboembolism in these patients, the use of heparin is also considered necessary, followed by warfarin in those with left ventricular ejection fractions of less than 35%. Although PPCM shares many features with other forms of nonischemic cardiomyopathy, an important distinction is that women with PPCM have a higher rate of spontaneous recovery of ventricular function. However, in a single centre prospective study of 100 South African patients with the condition, 15% died and only 23% recovered normal left ventricular function after 6 months of treatment, despite optimal medical therapy with ACE inhibitors and β blockers.[23] In a longer follow-up study of prospectively identified patients in Haiti over 5 years, the mortality rate was also 15%, and 29 of 92 (31·5 %) recovered normal left ventricular function. Continuing improvement was observed in the second and third years after diagnosis, confirming that the recovery phase is not limited to the first 6–12 months.

Subsequent monitoring depends on response to treatment, and includes a follow-up echocardiogram in the first several weeks to confirm improvement of left ventricular systolic function. After that, an echocardiogram is indicated approximately every 6 months until recovery is confirmed or a plateau is reached. The best time to discontinue ACE-inhibitors or β blockers is unknown; however, one or both of these medications should be continued for at least 1 year. Where resources exist, left ventricular assist devices and heart transplantation may be utilized if necessary.[32,33] Limited studies have been done with the use of immunosuppressive drugs such as azathioprine and steroids and have shown mixed results. Use of these agents should be reserved pending further assessments and perhaps should be restricted to patients with biopsy-proven lymphocytic myocarditis in the absence of viral particles. Recent studies in some patients with idiopathic dilated cardiomyopathy suggest a prominent role for cardiotrophic viruses.[34] As noted previously, only one investigation to date has identified viral genomic material in endomyocardial tissue from patients with PPCM.[35] Hence, PCR testing for a range of cardiomyotropic viruses could become important in the investigation of patients who do not improve in the early weeks following diagnosis. Aside from their haemodynamic benefits, the angiotensin-converting enzyme inhibitors, beta-blockers, and angiotensin-receptor blockers may have an additional benefit in dampening an over-active immune system that plays a role in the basic path physiology of PPCM. Given the potential inflammatory nature of PPCM there may also be a role for other immunomodulatory therapy. A prospective study of 59 consecutive women with PPCM reported a significant reduction in the inflammatory marker TNFα and improved outcome in patients receiving the immunomodulating agent pentoxifylline in addition to conventional therapy.[36]

## CONCLUSION

Peripartum cardiomyopathy (PPCM) is a rare cardiomyopathy of unknown cause which is a serious threat to life. PPCM remains a diagnosis of exclusion and Echocardiography provides clues to Diagnosis in a symptomatic patient. Decompensated PPCM needs a multidisciplinary approach and hence is a serious challenge in the Emergency Department as well as in the hospital.
